# Observation of Complex Conjugate Roots and Resonant Behavior in Quasi-Solid Supercapacitors as an Indication of Its Electrochemical instability

**DOI:** 10.1021/acsomega.4c10412

**Published:** 2025-03-20

**Authors:** Waldo Roberto Gallegos-Pérez, Asiel N. Corpus-Mendoza, Ana Karina Cuentas-Gallegos

**Affiliations:** † Instituto de Energías Renovables - Universidad Nacional Autónoma de México (UNAM), Priv. Xochicalco S/N, C.P. 62580 Temixco, Morelos, México; ‡ Consejo Nacional de Ciencia y Tecnología, Av. Insurgentes Sur 1582 Col. Crédito Constructor, Demarcación Territorial Benito Juárez, C.P. 03940 Ciudad de México, México; § Centro de Nanociencias y Nanotecnología, Universidad Nacional Autónoma de México, Km 107 Carretera Tijuana-Ensenada, Ensenada, B.C. C.P. 22800, México

## Abstract

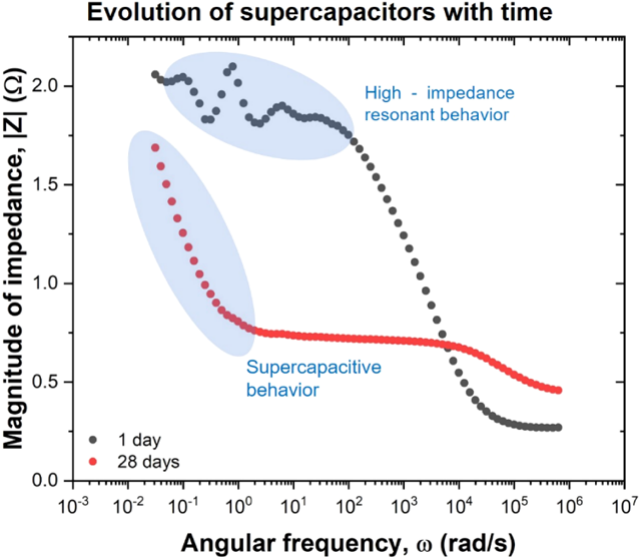

Quasi-solid supercapacitors are promising electrochemical devices for energy storage applications due to their high-power density, long life cycle, and environmental benefits. However, their electrochemical performance can change over time as a result of interactions between the electrodes and electrolyte, as well as the fabrication process. In this study, the electrochemical behavior of quasi-solid supercapacitors with activated carbon electrodes immersed in 4 M H_2_SO_4_ poly(vinyl alcohol) electrolyte for periods of 10 min and 24 h were investigated. Initial measurements show a lack of energy storing properties in newly fabricated devices, which improve with the aging time, as observed in cyclic voltammetry and charge–discharge cycles. Anticlockwise arcs and resonant peaks were observed in Nyquist and Bode plots, respectively, and were modeled by introducing complex conjugate roots and a damping factor ξ in the transfer function of the electronic equivalent circuit. This unfavorable behavior disappeared after 14 days in devices with shorter immersion times. On the other hand, the effects persisted in devices with longer immersion times even after 28 days. The stability of quasi-solid supercapacitors is thus demonstrated to be linked to complex conjugate roots and resonant behavior in impedance spectroscopy.

## 1 Introduction

Energy storage is a key challenge in the development of sustainable and efficient technologies, such as renewable energy, electric vehicles, smart grids, and portable devices. Among the different energy storage systems, electrochemical devices like supercapacitors (Scs) have gained significant attention due to their high-power density, long cycle life, and environmentally friendly design. Unlike batteries, Scs offer advantages such as the ability to undergo 10,000 to 100,000 charge–discharge cycles,^1,2^ good energy storage capacity (4–95 Wh kg^–1^),^3,4^ a wide operating temperature range, low maintenance cost, and the use of eco-friendly materials. These features make Scs ideal for applications like regenerative braking, power backup, peak shaving, and hybrid systems. They are also well-suited for emerging technologies like the Internet of Things (loT), sensors, and wearable electronics, where low energy consumption, high reliability, and long lifetime are essential. Therefore, improving Scs is important for their future role in energy storage.

A key factor improving the performance of Scs is the electrolyte, which provides the ionic conductivity and determines the electrochemical voltage window of the device. Quasi-solid electrolytes like poly(vinyl alcohol) (PVA) with sulfuric acid (H_2_SO_4_), offer advantages such as chemical stability, good ionic conductivity, low volatility, and a wide operating temperature range.^5,6^ Additionally, quasi-solid electrolytes can enhance the mechanical stability and safety of Scs compared to liquid electrolytes. Another important component of Scs is the electrode material, which affects the device’s capacitance, resistance, and energy storage performance. For this, carbon-based electrodes such as reduced graphene oxide (rGo), carbon fibers (CF), carbon nanotubes (CNTs) and activated carbon (AC) are usually preferred due to their high surface area, high porosity, low cost and wide availability.^7,8^ Some recent advances include the development of anode materials based on transition metal chalcogenides,^9−11^ such as CoSe_2_ embedded in N-dope carbon nanocubes^12^ to improve the cycling stability and diffusion kinetics of Li-ion Scs,^13−16^ microcrystalline hard carbon in a house-of-cards framework to shorten the diffusion path of Li-ion,^17−21^ and the flower-like heterostructure made of ZnO nanoparticles decorated with NiFe/CNT-rGo to obtain a dual electric double layer and pseudocapacitive behavior.^22,23^ Therefore, it is important to investigate carbon-based materials and optimize the electrode fabrication process and to consider factors such as electrode immersion time in the electrolyte, thickness, mass, and area, to improve the SC performance.

After assembly, supercapacitors are often evaluated using electrochemical impedance spectroscopy (EIS) to understand the electrochemical processes within the device. These processes are typically modeled using an electronic equivalent circuit (EEC) with passive components like resistors, capacitors, and constant phase elements. However, dynamic interactions between the electrode materials and the electrolyte can sometimes lead to unexpected impedance profiles, such as anticlockwise arcs and resonant peaks in Nyquist and Bode plots, respectively, These unusual effects are explained by hidden negative differential resistances or complex impedances.^24^ Although rarely reported in supercapacitors, similar behavior has been observed during the oxidation of metal electrodes in acid solutions^25−30^ influenced by acid concentration.^31^

In this study, we investigate the electrochemical behavior of quasi-solid Scs using stainless steel and AC electrodes with 4 M H_2_SO_4_ PVA electrolyte, varying the electrode immersion time before device assembly. Initially, the devices show poor energy storing properties, and their Nyquist and Bode plots resemble those seen during metal oxidation. However, this behavior decreases over time in devices immersed for 10 min until they exhibit the expected EIS profile after 14 days. In contrast, devices immersed for 24 h still show resonant behavior even after 28 days of aging. These findings indicate that the immersion time of AC electrodes in PVA-H_2_SO_4_ viscous electrolytes affects the interfacial contact and pore filling of the electrodes, and that longer immersion times do not necessarily improve the performance of quasi-solid Scs. Additionally, an EEC and transfer function was developed to explain the appearance and disappearance of complex roots in the Bode plots.

## 2 Experimental Section

### 2.1 Fabrication of Scs

A magnetic stirrer was used to mix 70 wt % AC as the active material (Norit DLC), 20 wt % conductive carbon (super P), 10 wt % Teflon and ethanol. The mixture was heated at 60 °C until it formed a black plastic-like dough, which became a semihard, powdery substance once the ethanol evaporated. To make the substance pliable, 4 drops of ethanol were added to 15 mg of the powder. The mixture was then spread onto a 1 cm^2^ stainless steel mesh (Aisi 316L 250 wire, 250 μm) using a spatula to create the electrode. The final AC electrodes were dried on a hot plate at 60 °C for 30 min, achieving an active mass of 15 mg/cm^2^.

**Figure 1:**
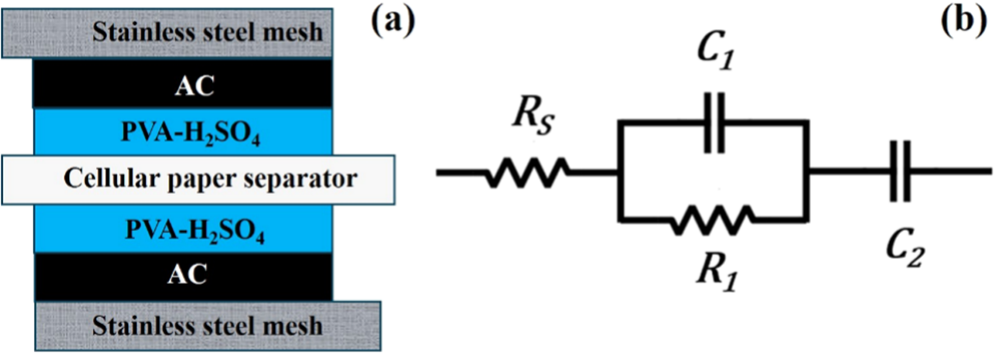
(a) Cross-section of a symmetrical supercapacitor (Sc) with quasi-solid electrolyte (PVA-H_2_SO_4_) and activated carbon (AC) Scs. (b) Ideal electronic equivalent circuit with a series resistance *R*_s_, polarization resistance *R*_1_, electrodes capacitance *C*_1_, and main capacitance *C*_2_.

The viscous or quasi-solid electrolyte was prepared by dissolving 1.5 g of PVA in 7.8 mL of water (half the total amount needed to prepare the acid electrolyte with the desired molar concentration) while stirring at 500 rpm. Then, 4.3 mL of concentrate H_2_SO_4_ was mixed with the remaining 7.8 mL of water and added to the PVA solution to achieve a final volume of 20 mL of 4 M of H_2_SO_4_. This concentration was chosen since it provided the maximum capacitance in our previous study.^32^ Then, the mixture was stirred for 5 min at 1100 rpm and 65 °C to form a semitransparent viscous gel. Two AC electrodes were then immersed in the PVA-H_2_SO_4_ 4 M electrolyte for either 10 min or 24 h, followed by 10 min of rest at room temperature to remove excess electrolyte. A piece of cellulose paper was placed between the electrodes as a separator to prevent short circuits. The SC was then assembled and sealed using adhesive tape and hot silicone, as shown in the cross-section in Figure 1a.

### 2.2 Electronic Equivalent Circuit

The ideal electronic equivalent circuit of a Sc consists of an ionic resistance, *R*_S_ from the electrolyte, along with two parallel elements: *R*_1_, which represents the polarization resistance, and *C*_1_, which accounts for the small capacitance of the electrodes. The main capacitance (*C*_2_) of the supercapacitor is where the energy is stored, as shown in Figure 1b. The total impedance (*Z*_T_) is described by eq 1, and the circuit can also be represented with the transfer function in eq 2 or as a factorized transfer function in eq 3. Here, *s* is the complex frequency, defined as *s* = *j*ω *= j*2π*f*, where ω is the angular frequency in rad s^–1^, *f* is the frequency in Hertz, and *j* = √−1 is the imaginary unit.
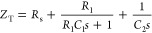
1
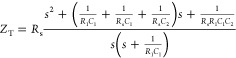
2
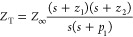
3

The previous equations assume ideal capacitors, but a more accurate model can be developed by treating capacitors as constant phase elements (CPE). However, for the sake of simplicity, only ideal capacitors will be considered in this analysis.

### 2.3 Characterization of Quasi-Solid Scs

Cyclic voltammetry (CV), galvanostatic charge–discharge (GV) cycles, and EIS of Scs were conducted using a VMP-300 potentiostat from BioLogic Science Instruments. The voltage window of each Sc was determined with CV at scan rates of 5, 10, 20, and 50 mV s^–1^. GV was performed using specific currents of 0.1, 0.25, 0.5, and 1 A g^–1^. For EIS, a sinusoidal wave with a 10 mV amplitude, a DC voltage of 0 V, and frequencies ranging from 100 kHz to 5 mHz were applied. Finally, the specific capacitance (*C*_T_, F g^–1^), specific energy density (*E*, Wh kg^–1^), and specific power (*P*, W kg^–1^) of the best Scs were calculated using eqs 4 to 6.^30−33^
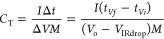
4
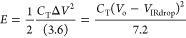
5
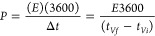
6where *I* is the constant current used for charging and discharging in thee used in GV, *t_Vf_*, the time at the end of discharge, *t*_*Vi*,_ the time when the maximum voltage is reached, *V*_o_, the voltage window, *V*_IRdrop_, the voltage drop during discharge, and *M*, the mass of both active electrode (70% of AC). The devices were horizontally stored in a drawer inside a laboratory kept at 25–30 RH% and 25–30 °C through the characterization period and taken out just during the measurements.

## 3 Results and Discussion

**Figure 2:**
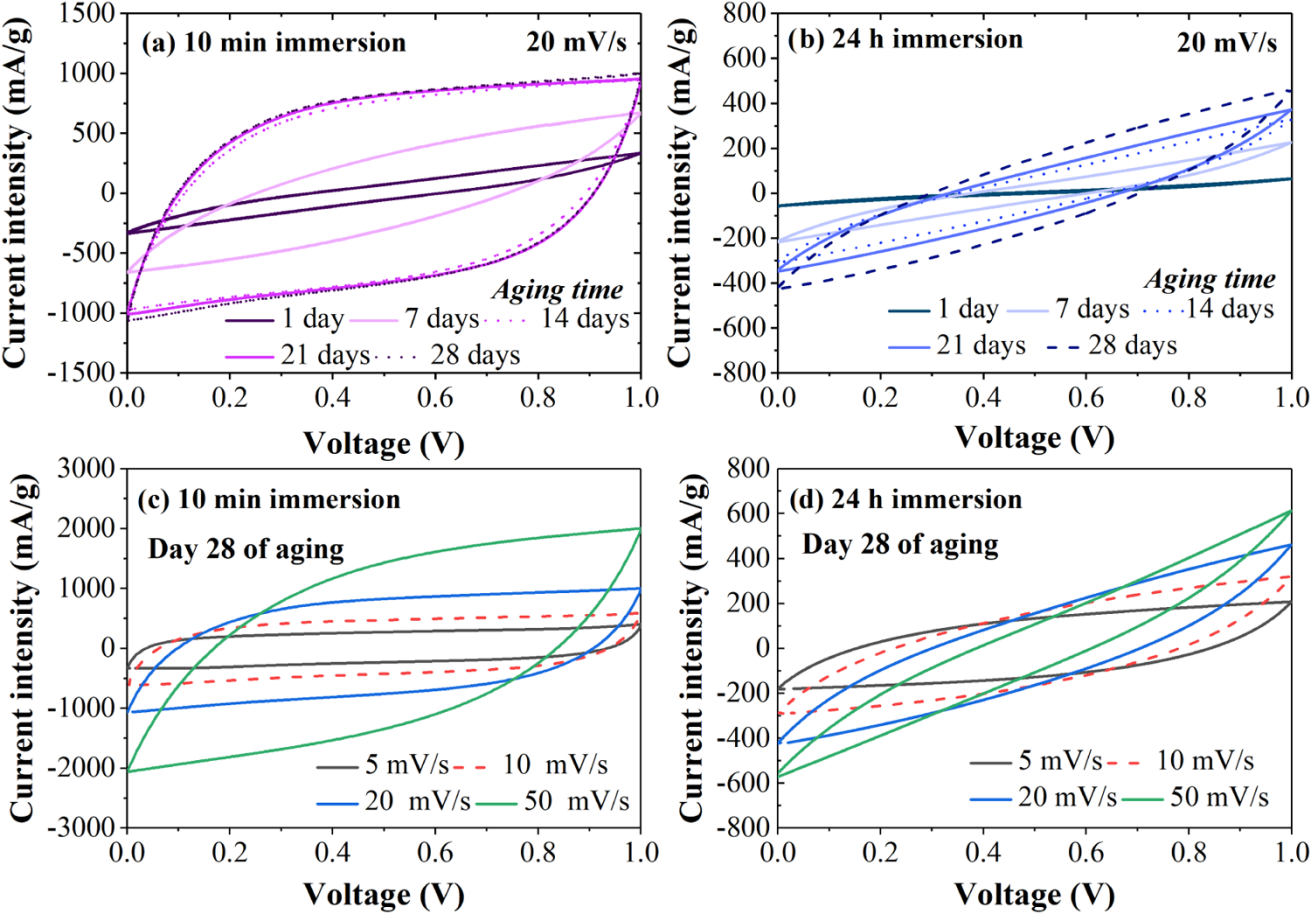
(a) CV Curves of 10 min and (b) 24 h immersion AC Scs at 20 mV s^–1^ for 28 days of aging. CV curves of (c) 10 min and (d) 24 h immersion Scs using a scan rate from 5 to 50 mV s^–1^ after 28 days of aging.

Figure 2a,b show the CV curves of the AC SCs immersed for 10 min and 24 h, respectively. Both devices initially show a resistive behavior at a scan rate of 20 mV s^–1^. However, over time, the Scs immersed in the electrolyte for 10 min begin to show a semirectangular CV profile, which is characteristic of the double-layer storage mechanism occurring at the electrode–electrolyte interface.^33,34^ In contrast, the Scs immersed for 24 h show little change in their CV profile even after 28 days of aging. This improvement over time for the 10 min immersed device may be related to a diffusion process, possibly due to the presence of PVA in the electrolyte acting as a permeable barrier. After 28 days of aging, the Scs immersed for 10 min demonstrate a semirectangular profile for scan rates between 5 and 50 mV s^–1^, while those immersed for 24 h do not exhibit energy storage properties, as shown in Figure 2c,d. This indicates a clear advantage in both performance and fabrication time for Scs with electrodes immersed for only 10 min.

**Figure 3:**
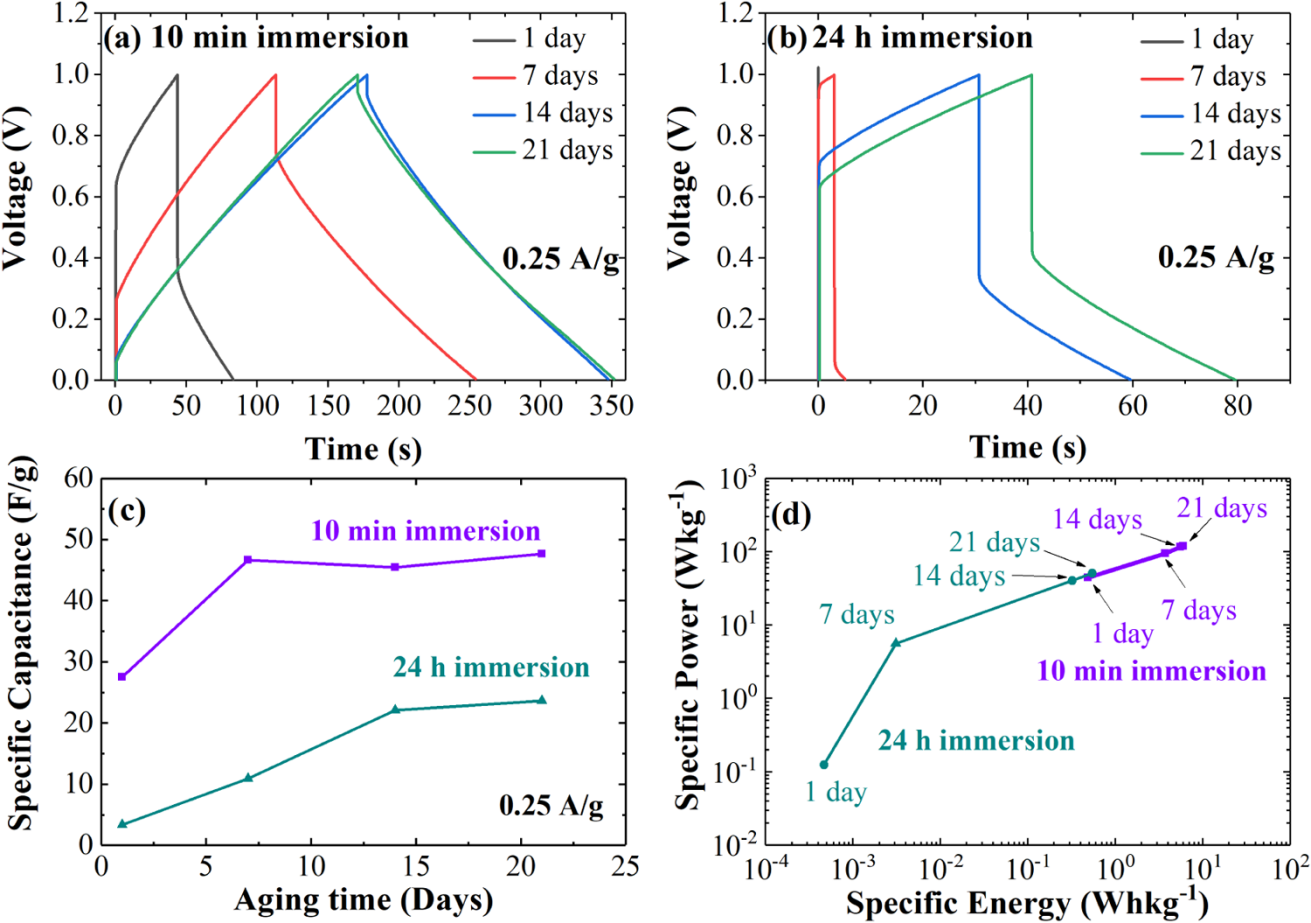
Charge–discharge (GV) curves performed using 0.25 A g^–1^ current density of AC Scs with (a) 10 min and (b) 24 h immersion time. (c) Specific capacitance, and (d) Ragon plot in terms of specific power vs energy for AC Scs.

Figure 3a,b show the GV plots of devices immersed for 10 min and 24 h, measured at 0.25 A g^–1^, respectively. The triangular charge–discharge cycle becomes more prominent in the devices immersed for 10 min as they age. In contrast, the devices immersed for 24 h show a sharp increase and decrease in voltage before the controlled charge and discharge process, which indicates a highly resistive behavior. After 21 days of aging, the device with a 10 min immersion reaches a maximum voltage of 1 V, followed by a small *V*_IRdrop_ and then a discharge from 0.9 V over 181 s. In comparison, the 24 h immersion device experiences a significant *V*_IRdrop_ to 0.4 V, followed by a discharge in 38.53 s. The triangular profiles shown in Figure 3a at 14 and 21 days confirm the good reversibility of the adsorption/desorption process in the 10 min AC Sc. Figure 3c shows the specific capacitance, *C*_T_, calculated from eq 4 for both devices as a function of aging time. The 10 min AC Sc shows an initial *C*_T_ of 27.49 F g^–1^, which increases to 47.62 F g^–1^ by the 21st day. In contrast, the 24 h AC Sc has an initial *C*_*T*_ of 3.24 F g^–1^, which only increases to 23.63 F g^–1^. Figure 3d shows the specific energy, *E*, and specific power, *P*, calculated from eqs 5 and 6, revealing that the 10 min AC Sc improves over time, achieving a power of 118 Wh kg^–1^ and an energy of 5.97 W kg^–1^. In comparison, the 24 h AC Sc has values of 50 Wh kg^–1^ for power and 0.54 W kg^–1^ for energy after 21 days. These results indicate that allowing the Scs to age for several days can enhance their performance, as demonstrated by the semirectangular CV curves, triangular charge and discharge profiles, and improved electrochemical values of *C*_T_, *P* and *E*, particularly for Scs assembled with electrodes immersed in the electrolyte for just 10 min.

**Figure 4:**
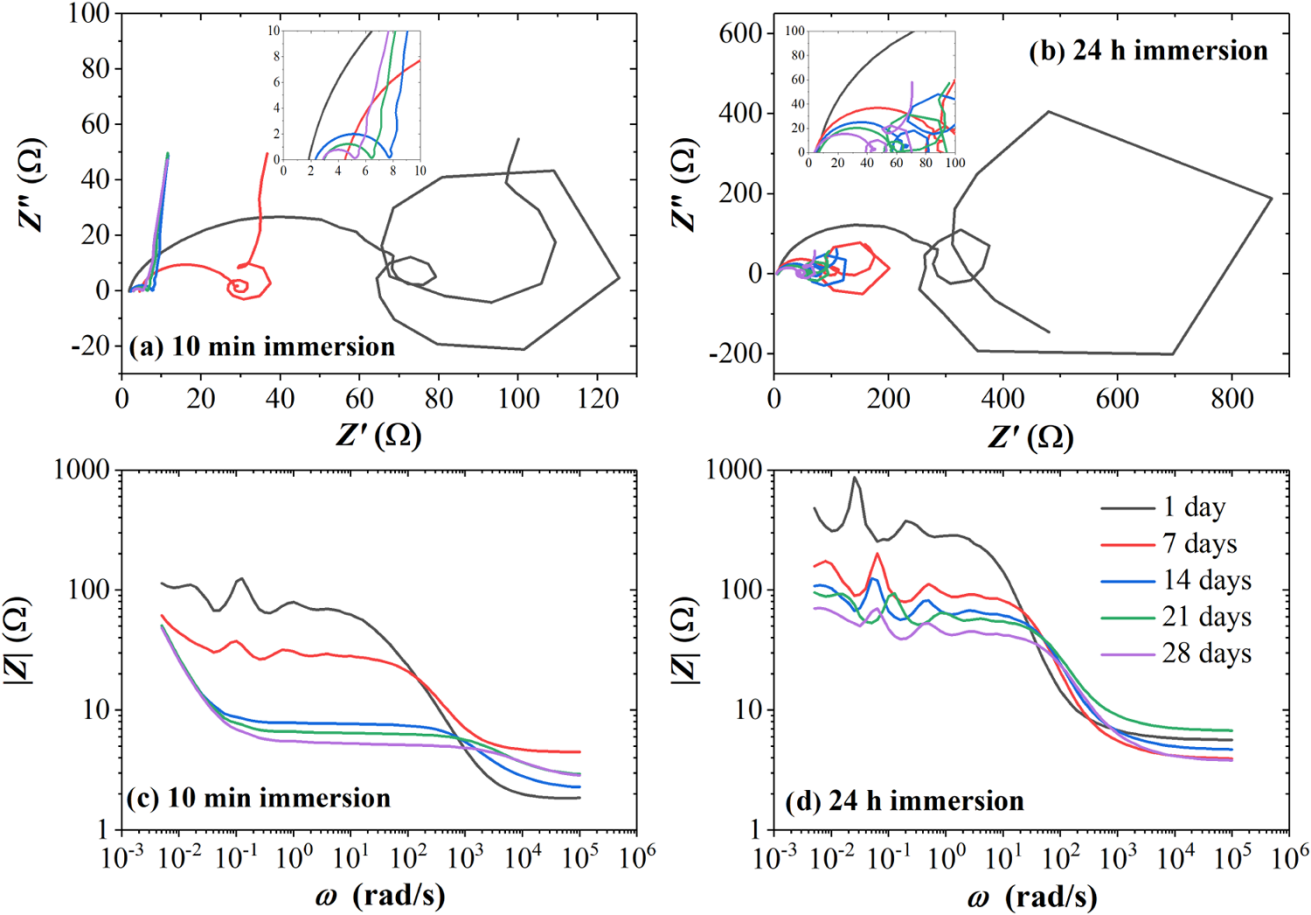
(a) and (b) Nyquist diagrams of 10 min and 24 h immersion Scs, respectively, from 5 mHz to 100 kHz. Bode plots for (c) 10 min and (d) 24 h immersion Scs.

Figure 4a,b show the evolution of the Nyquist diagrams for the Scs immersed for 10 min and 24 h, respectively. The Nyquist plots after several days of aging approach the expected behavior described by eqs 1 and 2, which include an ionic resistance observed at very high frequencies, a small clockwise arc transitioning from high to low frequencies due to polarization resistance and electrode capacitance, and a straight vertical line in the low-frequency range representing the main capacitance of the supercapacitor. In contrast, the Nyquist plots during the initial days show anticlockwise loops before the straight vertical line reveals as the frequency decreases. These anticlockwise loops have been modeled in the literature as hidden negative differential resistances, indicating inductive behavior and causing the Nyquist plot to display positive values on the imaginary axis. However, unlike previous reports of negative resistance, our measurements do not reach negative values on the real axis. Similarly, negative resistance and inductive behavior at low frequencies have been observed during the electro-oxidation of methanol while using a Pt-based catalyst^35^ but for intermediate potentials applied due to a decrease of CO during the reaction.^36−39^ This suggests that the negative resistance effect is related to the CO residues that result from our AC electrodes when in contact with the acid electrolyte. As a result, the AC electrodes of devices immersed for 24 h degrade more than those immersed for only 10 min due to an increased pore wetting, which then reveal the low-frequency artifacts observed. Furthermore, the Bode plots of the 10 min Scs also evolve with aging. The magnitude of the impedance |*Z*| in the medium-frequency range decreases over time, revealing a descending slope as a function of ω in the lower-frequency regime, which is expected behavior for a capacitor. This observation aligns with the improvement in energy storage capabilities noted over time in the CV, and charge–discharge GV, tests. However, an unusual resonant behavior of |*Z*| is observed during the initial days of the 10 min Sc, which then disappears with aging. A similar behavior is also noted for the 24 h Sc, although it starts at a higher |*Z*|, does not achieve the initial descending slope, and retains its resonant behavior over time. Such resonant behavior is often observed in four-terminal EIS measurements as a result of the combination of sample impedance, experimental media, geometry and position of test electrodes, internal impedance of the measurement equipment, and capacitive coupling of the signal lines,^40^ however, this is often avoided by using two-terminal measurements of solid devices as in our case.

We also observe that the Nyquist, Bode, and Ragon plots of the 24 h Sc after 28 days of aging are similar to the initial plots of the 10 min Sc, therefore, it may be possible that the 24 h Sc may continue to improve for further days.

**Figure 5:**
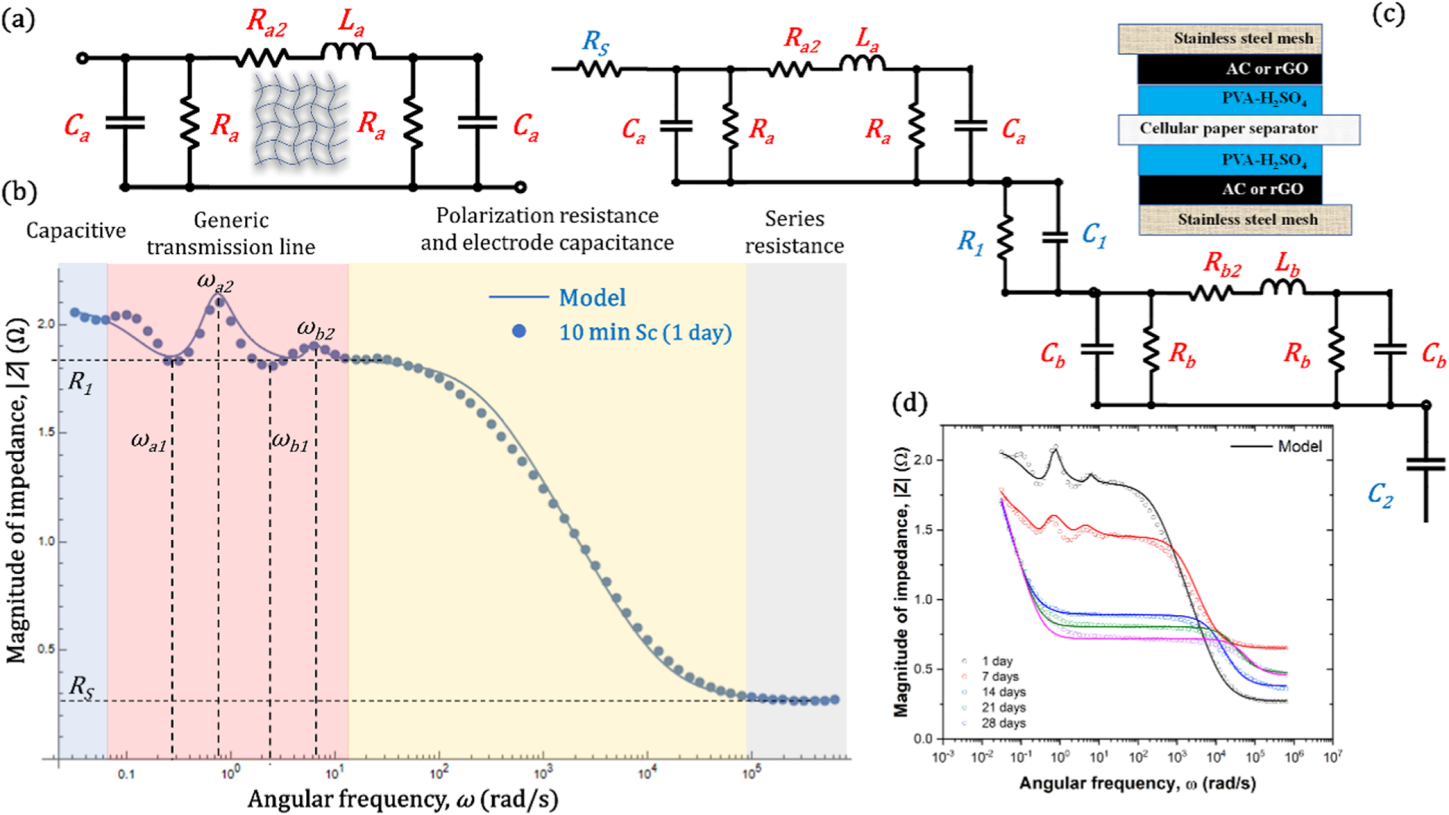
(a) Generic transmission line equivalent circuit. (b) Effect of the EEC elements in the |*Z*|. (c) Complete EEC to model |*Z*| of the supercapacitor. (d) EEC model fitting of |*Z*| for different aging days.


One method to reproduce the resonant behavior of |*Z*| in the Bode plot is by using a generic transmission line, as illustrated in Figure 5a. This model includes resistive (*R*_a_, *R*_a2_) and capacitive (*C*_a_) effects attributed to the porous AC, which is often represented as a capacitive transmission line. Additionally, the generic transmission line also considers the possibility of inductive (*L*_a_) effects resulting from the stainless steel mesh used as a current collector. The impedance of the generic transmission line is calculated to yield the transfer function shown in eq 7, which can be simplified to include two terms with complex conjugate roots, one in the denominator and one in the numerator, as shown in eq 8. This formulation, which can result from various circuits, facilitates the identification of ω_1_ and ω_2_ as the angular frequency values corresponding to the valleys and peaks, respectively, as shown in Figure 5b. Also, the damping ratio (ξ ≥ 0) regulates the amplitude of the peaks, with ξ = 0 representing an underdamped system that produces the sharpest peaks, while higher values of ξ decrease the amplitude. The Bode diagrams of the 10 min Sc after 1 day of aging exhibit 2 distinct peaks and valleys in the low-frequency range, attributed to the symmetrical structure of the Sc, which has 2 stainless steel and AC electrodes. Slight variations between each electrode may cause shifts in the frequency (ω) of their peaks. Therefore, the complete EEC is shown in Figure 5c, where *C*_2_ represents the main capacitance of the device, *R*_1_ and *C*_1_ influence the descending slope in the medium to high frequencies, and *R*_s_ indicates the series resistance observed at significantly high frequencies. This results in the total impedance |*Z*_Total_| presented in eq 9. As the device ages, the relevance of the transmission line terms diminishes, leading to the disappearance of the resonant behavior, while the impact of the main capacitance becomes more pronounced, as modeled in Figure 5d.

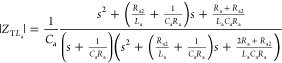
7
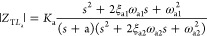
8
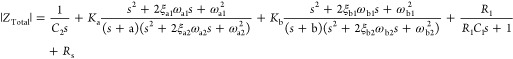
9

Since the value of *R*_1_ needed to fit |*Z*| is highest for devices immersed in electrolyte for 24 h, it is likely that the excess of PVA increases |*Z*| during immersion. Additionally, the term 2ξ_a_ω_a_, expressed as *R*_a2_/*L*_a_ + 1/*C*_a_*R*_a_ = (*R*_a2_*C*_a_*R*_a_ + *L*_a_)/*L*_a_*C*_a_*R*_a_ in the transfer function, causes the resonant peaks when ξ < 1. This occurs when *R*_a2_*C*_a_*R*_a_ + *L*_a_ < 2ω_a_*L*_a_*C*_a_*R*_a_, indicating the presence of resonant behavior. This suggests the possibility of unusual values for *L*_a_ and *C*_a_, or even negative values for *R*_a2_, especially if ω_a_ is small. In any case, the value of ξ is expected to increase as the resonant behavior decreases, eventually reaching ξ > 1 in aged devices, at which point the peaks disappear entirely. When this occurs, the Nyquist plot no longer shows loops or anticlockwise arcs, removing the need to consider inductance or negative resistance in the model.

## 4 Conclusions

Supercapacitors were fabricated by immersing activated carbon electrodes with stainless steel mesh as current collectors in H_2_SO_4_ PVA electrolyte for 10 min or 24 h. Initially, both devices showed highly resistive behavior. However, after 14 days of aging, the 10 min devices began to show energy storage properties, while the 24 h devices showed minimal improvement even after 28 days. Galvanostatic charge–discharge measurements revealed that, after 28 days, the10 min devices sustained a full discharge from 0.9 V in 181.11 s, while the 24 h devices discharged from 0.4 V in 38.53 s. Impedance spectroscopy indicated the presence of inductive loops and anticlockwise arcs in the Nyquist plot of freshly prepared devices, which gradually disappeared with aging. This was reflected in the resonant behavior observed in the Bode plot at medium frequencies, which could be modeled using a transmission line incorporating a damping factor ξ and complex conjugate roots in the impedance transfer function. The model suggests the involvement of unusual inductance values or hidden negative resistance during early aging stages. The resonant behavior vanished in the 10 min devices after 28 days, whereas the 24 h devices still resembled newly prepared 10 min supercapacitors, suggesting further improvement with time. The resonant behavior and the initial lack of energy storage is attributed to degradation of the carbon-based electrodes when in contact with acid electrolytes. These insights provide valuable guidelines to understand the evolution and optimization of electrodes to potentially enhance the performance of carbon-based supercapacitors for practical energy storage applications.
